# Histopathological findings in the brain decades after cingulotomy: An autopsy case

**DOI:** 10.1002/pcn5.70197

**Published:** 2025-08-25

**Authors:** Youta Torii, Shuji Iritani, Hiroshige Fujishiro, Hirotaka Sekiguchi, Ayako Miwa, Ryuichi Koizumi, Mari Yoshida, Yasushi Iwasaki, Kazumi Sasada, Masashi Ikeda

**Affiliations:** ^1^ Department of Psychiatry Nagoya University Graduate School of Medicine Nagoya Aichi Japan; ^2^ Department of Psychiatry Moriyama General Mental Hospital Nagoya Aichi Japan; ^3^ Brain Research Institute Okehazama Hospital Fujita Kokoro Care Center Toyoake Aichi Japan; ^4^ Institute for Medical Science of Ageing Aichi Medical University Nagakute Aichi Japan; ^5^ Department of Neurology and Stroke Medicine Graduate School of Medicine, Yokohama City University Yokohama Kanagawa Japan

Cingulotomy is a psychosurgical procedure developed as an alternative to prefrontal leucotomy to treat various psychiatric disorders to avoid associated complications.^1^ To the best of our knowledge, psychosurgery has not recently been performed in Japan. However, cingulotomy has been reported as a treatment option for limited conditions such as refractory obsessive‐compulsive disorder and intractable pain in some countries.^2^, ^3^ Traditionally, after detecting the cingulate gyrus using neuroimaging techniques, the anterior cingulate gyrus (ACG) tissues are ablated through burr holes in the skull.^1^

In autopsy cases of prefrontal leucotomy, chronic axonal damage resulting from the procedure can lead to the accumulation of phosphorylated tau (p‐tau) in neurons, astrocytes, and cell processes, indicating a pathology similar to chronic traumatic encephalopathy (CTE).^4^ Furthermore, it has been reported that this p‐tau accumulation may progress to connected regions via neuronal circuits over time.^5^ However, to our knowledge, the long‐term impact of cingulotomy on the brain tissue has not yet been reported. We herein report an autopsy case of a patient with schizophrenia who underwent cingulotomy several decades earlier.

The male patient developed schizophrenia at approximately 15 years of age. He exhibited disorganized behavior, agitation, violent acts, refusal, silence, and autistic behavior. The patient underwent cingulotomy at 21 years of age. Postoperatively, his verbal communication improved, and his violent behavior gradually subsided. No significant behavioral problems were observed during middle age. However, in his 60 s, he developed progressive memory loss that interfered with daily life and was diagnosed with Alzheimer's disease (AD). The patient ultimately died at 70 years of age due to aspiration pneumonia.

The whole brain weighed 1330 g. Macroscopic evaluation revealed tissue defects in the corpus callosum and the ACG (Figure 1a,b). Microscopically, Klüver‐Barrera staining showed thinning of the interrupted cingulate gyrus and pallor of the surrounding myelin (Figure 1c,e). The area of myelin pallor corresponded well with the white matter gliosis observed with Holzer staining (Figure 1d,f). Anti‐phosphorylated neurofilament H‐positive neuronal soma was observed in the ACG (Figure 1g,h) and was widely distributed across the medial and lateral frontal cortices (Figure 1i), reflecting impairment of axonal transport.^6^ Fragmented and thickened axons were present in the white matter of the surgical site (Figure 1j).

**Figure 1:**
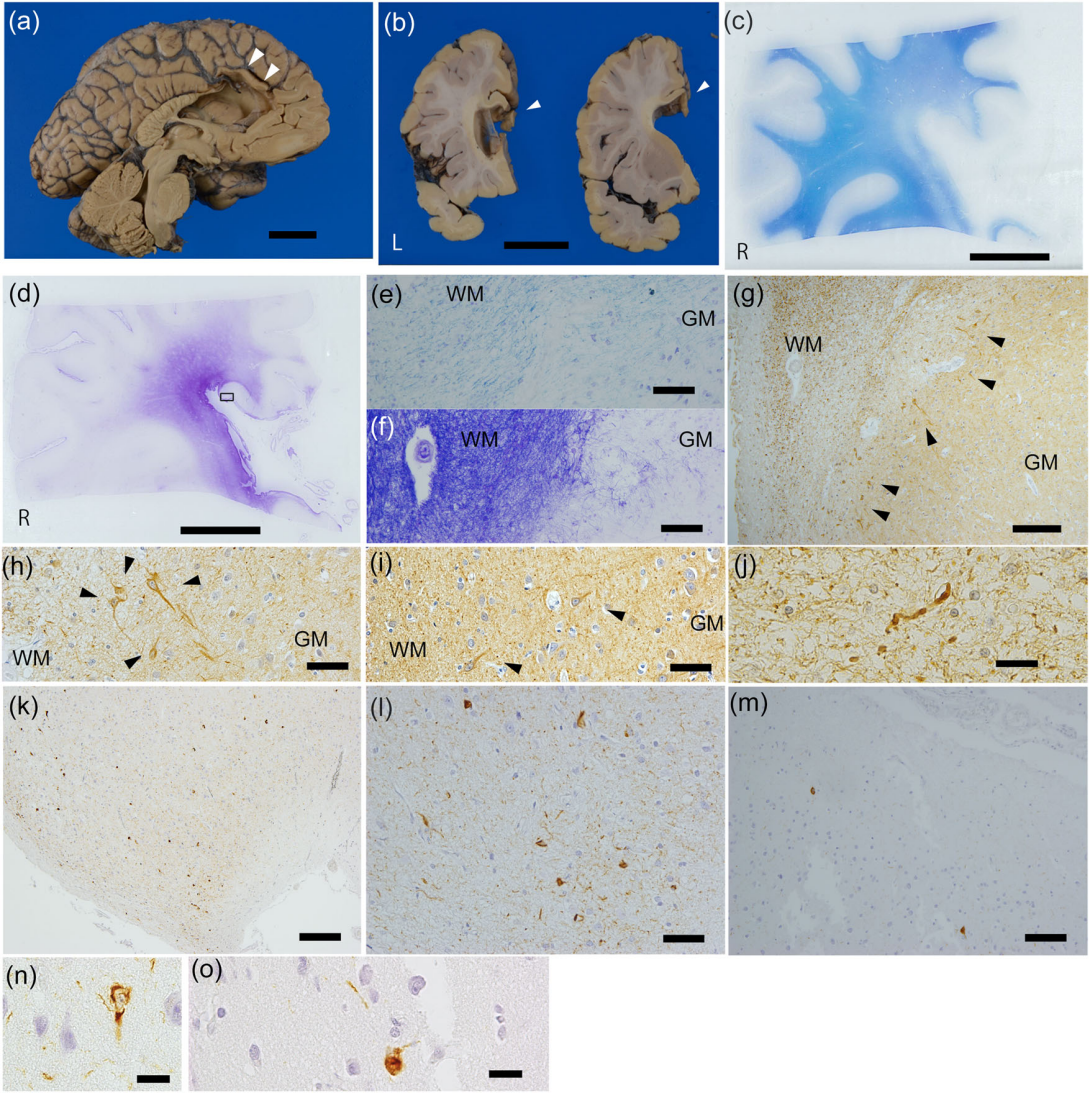
Neuropathological findings. (a) Medial surface of the left cerebral hemisphere. (b) Coronal section of the left cerebrum. Arrowheads in (a, b) indicate tissue defects in the corpus callosum and anterior cingulate gyrus. No significant atrophy of the cerebrum was noted. The basal ganglia, brainstem, and cerebellum showed no remarkable changes. Myelin pallor is shown with Klüver‐Barrera staining (c, e), and gliosis in the white matter around the cingulate gyrus is demonstrated with Holzer staining (d, f). The rectangle in (d) indicates the area shown in (k). Anti‐phosphorylated neurofilament H (pNF‐H)‐positive neuronal soma (arrowhead) in the anterior cingulate gyrus (g, h) and the superior frontal gyrus (i), which was particularly strongly observed in the deeper cortical layers. (j) Fragmented and thickened axons with anti‐pNF‐H immunostaining in the white matter around the cingulate gyrus. Axonal spheroids were not observed. The accumulation of p‐tau is shown at the severed end of the anterior cingulate gyrus with anti‐p‐tau immunostaining (k, l, n). The accumulation of p‐tau is also shown in the region distant from the severed end, in cingulate gyrus (m, o). (n) and (o) indicates the p‐tau accumulation of neurons, which was observed particularly in cortical layers II and III. Scale bars: 3 cm (a, b), 10 mm (c, d), 200 μm (g, k), 100 μm (m) 50 μm (e, f, h, i, l), 20 μm (j, n, o). GM, gray matter; WM, white matter.

At the severed end of the ACG, p‐tau accumulation was observed in many neurons and neurites (Figure 1k,l,n) as neurofibrillary tangles (NFTs) and neuropil threads. Sparse p‐tau‐positive neurons and neurites were also scattered in regions of the ACG distant from the severed end (Figure 1m,o). However, pathological features of CTE, such as p‐tau accumulation around small blood vessels, as reported in prefrontal leucotomy cases,^4^, ^5^ were not observed, and thus did not fulfill the neuropathological criteria for CTE.^7^

Moderate p‐tau accumulation, such as in NFTs or neuropil threads, was observed in the transentorhinal cortex, and mild accumulation was also found in the entorhinal cortex, hippocampal CA1 region, amygdala, occipitotemporal gyrus, locus coeruleus, and periaqueductal gray, corresponding to Braak stage III. However, there was no p‐tau accumulation in the prefrontal cortex and anterior thalamic nuclei, which are assumed to be regions connected to the ACG.^8^ All p‐tau‐positive lesions, including the ACG, exhibited positivity for Gallyas‐Braak staining. Diffuse plaques corresponding to Thal Phase 1 were observed. Neuritic plaques were absent. “ABC score” for neuropathological change is low. Aging‐related tau astrogliopathy (ARTAG) was not observed. A few anti‐phosphorylated transactive response DNA‐binding protein 43‐kDa‐positive neurons have been detected in the entorhinal cortex. α‐Synuclein‐positive structures were absent.

A postmortem clinical record review confirmed that dementia in this case met the criteria for all‐cause dementia: the core clinical criteria outlined in the National Institute on Aging–Alzheimer's Association guidelines for the neuropathologic assessment of Alzheimer's disease. In terms of clinicopathological correlations, the pathological substrate of incident neurocognitive decline could not be identified using current pathological classifications, including those for CTE and AD. This finding was consistent with a previous finding that a subset of patients with schizophrenia develop neurocognitive decline without coexisting dementia‐related pathology.^9^ The cause of this discrepancy remains unknown.

The distribution of p‐tau accumulation observed in this case overlapped multiple regions that were anatomically connected to the ACG.^8^ However, except for the abundance observed at the ACG transection site, the distribution was within the range expected for Braak stage III. Accordingly, although p‐tau at the site of ACG transection was assumed to result from cingulotomy, we found no evidence supporting the progression of p‐tau across brain regions via neuronal circuits.

Although there may be differences in p‐tau accumulation and its development among surgical techniques, the underlying factors remain unknown. The long‐term effects of psychosurgery on p‐tau accumulation are not well understood. In addition, there is insufficient systematic research on the long‐term cognitive changes following cingulotomy. Active and systematic research is needed as these psychosurgeries continue to be performed under certain conditions.

One limitation associated with our case report is that the cingulotomy procedure used was an older technique than that currently applied.

## AUTHOR CONTRIBUTIONS

Clinical data were evaluated by Youta Torii, Ayako Miwa, Kazumi Sasada, Shuji Iritani, Hiroshige Fujishiro, and Hirotaka Sekiguchi. Neuropathological evaluations were conducted by Youta Torii, Ryuichi Koizumi, Mari Yoshida, and Yasushi Iwasaki. The manuscript was written by Youta Torii, Hiroshige Fujishiro, Hirotaka Sekiguchi, Shuji Iritani, and Masashi Ikeda. All the authors approved the final manuscript.

## CONFLICT OF INTEREST STATEMENT

The authors declare no conflicts of interest.

## ETHICS APPROVAL STATEMENT

This study was approved by the Ethics Review Committee of the Nagoya University Graduate School of Medicine. The autopsy was performed in accordance with the “Corpse Autopsy and Preservation Law.”

## PATIENT CONSENT STATEMENT

After the patient's death, written consent for publication was obtained from the proxy.

## CLINICAL TRIAL REGISTRATION

N/A.

## Data Availability

Data sharing not applicable to this article as no datasets were generated or analysed during the current study.
